# What’s the Risk? Fearful Individuals Generally Overestimate Negative Outcomes and They Dread Outcomes of Specific Events

**DOI:** 10.3389/fpsyg.2019.01676

**Published:** 2019-07-30

**Authors:** Kristina M. Hengen, Georg W. Alpers

**Affiliations:** Department of Psychology, School of Social Sciences, University of Mannheim, Mannheim, Germany

**Keywords:** anxiety, fear, risk, overestimation, cognitive bias

## Abstract

Although it is an adaptive mechanism that danger usually elicits fear, it seems that fearful individuals overestimate the danger associated with their feared objects or situations. Previous research has not systematically distinguished between the estimated risk of an encounter with fear-relevant stimuli and the expected unpleasant outcomes of such encounters. Furthermore, it is not clear if biased risk perception is specific to an individual’s fear or generalized to all negative events. In an online-survey (*N* = 630) we assessed the estimated risk to encounter fear-relevant stimuli and the expectations of negative outcomes of such encounters. Items contained three domains (spiders, snakes, and everyday fear triggers). In regression analyses we examined the specific associations between fear and risk estimations. In addition, we compared subgroups with specific fears and low fearful individuals. While an individual’s fear score was not related to the estimated risk of an encounter with fear-specific stimuli, it was related to an overestimation of negative outcomes in all domains. The perceived risk of aversive outcomes was most pronounced for an individual’s specific fear. Furthermore, an individual’s specific fear was most predictive of the estimated risk of a negative fear-relevant outcome. Highly fearful individuals overestimate the risk of negative outcomes of fear-relevant encounters. Specifically, they dread outcomes of encounters with their feared object. Differentiating fear-relevant components of risk perception provides insights into the cognitions which may motivate maladaptive avoidance behavior.

## Introduction

In everyday life, we must often decide whether we accept or reject impending risk. Naturally, the way we evaluate and process different options plays an important role in our fear response (of a car crash, in this example).

In the literature, the close relationship between fear and information processing has long been considered an important topic in clinical psychology (e.g., Foa and Kozak, 1986; Beck and Clark, 1997). Anxiety not only results in biased information processing implicitly, e.g., attentional bias (Bar-Haim et al., 2007), but also in explicit biases in the evaluation of information, e.g., risk perception (e.g., Loewenstein and Lerner, 2003; Lerner et al., 2003). For example, more fear results in higher probability ratings of negative events, e.g., to fail in an exam (Butler and Mathews, 1987) and increased estimated risks related to terrorism in the wake of September 11th, 2001 (Lerner et al., 2003). The link between fear and heightened risk perception has informed the so called *risk as a feeling* hypothesis (Loewenstein et al., 2001) that highlights the role of emotions felt during and anticipated after the evaluation of different options.

To continue, fear-related anticipatory responses are generally adaptive and serve a beneficial function and reduce the risk of harm (Rosen and Schulkin, 1998). These reactions to fear-relevant events can also be described as a cognitive process oriented toward the future (Paulus and Stein, 2006) as they are typically focused on threat or the possibility of potentially negative events (Barlow et al., 1996). Assessing the probability of negative events that can occur in the future may therefore help to allocate cognitive resources efficiently. In line with this, the *Affect heuristic* (Finucane et al., 2000) states that affect serves as a cue for information processing. It builds an impression by consulting a pool of potential affective reactions to the stimulus item. This may facilitate many judgments and decisions, especially so when they are complex and mental resources are limited (Finucane et al., 2000; Slovic et al., 2005).

However, when risk estimates exceed the true likelihood or when the severity of the future event is exaggerated, this fear process may be maladaptive (Hofmann et al., 2008). Interestingly, many of the threats phobic individuals dread are not based on an objective risk (Gerdes et al., 2009). What is more, phobic individuals may have limited insight into the irrationality of their anticipatory responses (Jones and Menzies, 2000). Even outside of the feared situation, phobic individuals gave higher danger expectancy ratings of being bitten by a spider, and higher ratings for the injuries that would result from the bite.

This maladaptive risk evaluation has long been a cornerstone of cognitive models of anxiety disorders and has been assumed as a mediating variable between phobic fear and avoidance (McNally and Foa, 1987; Beck and Clark, 1997). In addition, it may imply unnecessary suffering and may block cognitive resources. Furthermore, excessive anticipatory responding is considered to limit extinction learning by triggering the avoidance of phobic stimuli (Öst, 1996).

In the laboratory setting, many studies show that fearful individuals exhibit a biased risk perception of aversive outcomes following fear-relevant stimuli; this was coined the covariation bias (Tomarken et al., 1989) (for a review see Wiemer and Pauli, 2016). In the first study of this kind, participants with high and low fear of spiders or snakes overestimated the probability of an aversive shock to co-occur with fear-relevant stimuli, although all stimuli-outcome combinations were equally likely to occur. More recent studies replicated this finding in socially anxious individuals who tended to overestimate the likelihood of an electric shock in conjunction with an angry face compared to a happy or neutral face (de Jong et al., 1998). Similar effects were found for fears of other animals (e.g., de Jong et al., 1995; Amin and Lovibond, 1997; Kennedy et al., 1997), fear of contamination (Olatunji et al., 2006), blood-injury fear (Connolly et al., 2009), and in panic-prone individuals (Pauli et al., 2001).

These findings have been extended to the *a priori* expectancy bias, i.e., exaggerated expectations of the stimulus-outcome contingency which occur independent of an actual experience: Fearful individuals overestimated the *a priori* likelihood of an aversive outcome after a fear-relevant stimulus (e.g., McNally and Heatherton, 1993; Cavanagh and Davey, 2000; van Overveld et al., 2010).

While these studies nicely demonstrate that the risk of aversive events are often exaggerated by fearful individuals in such experimental paradigms, it is less clear if this biased risk perception is (a) related to the encounter of fear-relevant stimuli or (b) related to the unpleasant outcomes such an encounter may have. A further limitation of the covariation bias literature is the artificial character of the design and, thus, its questionable ecological validity. For example, the covariation of fear-related pictures and electric shock may not be the same as the overestimation of the likelihood of running into a vicious dog on the street and being severely hurt by a bite.

A relatively small questionnaire study on risk estimations of fearful individuals in a variety of domains has not found distorted risk assessments in fearful individuals (Nesse and Klaas, 1994). Instead, all participants (patients with anxiety disorders and non-anxious participants alike) overestimated rare risks, underestimated risks of common events, and overestimated the relative risks of a threat to oneself. One reason for this unexpected finding could be that the encounter of fear-relevant events was confounded with the negative outcomes of these events in the assessment of this study.

Indeed, one study highlighted the importance to investigate two forms of risk estimations and not only to focus on the covariation bias that is thought to address negative outcomes (Aue and Hoeppli, 2012). These researchers focused on the encounters of fear-relevant stimuli and asked spider fearful and non-fearful individuals to rate the probability of encountering different animals (e.g., spiders, snakes, and birds) in different locations. Against expectations, fearful individuals compared to non-fearful individuals did not overestimate the likelihood of encountering any of the animals. Within the spider fearful group, individuals overestimated the likelihood of encountering a spider compared to the other animals. These findings give indirect insight into the distinctiveness between risk perceptions of encounters of fear-relevant stimuli and the negative outcomes of such encounters. However, the study lacks risk estimations of negative outcomes and thus is not able to draw further conclusions on the relation between the two components of perceived risk and their distinctiveness.

One investigation that examined the two biases together found that encounter and outcome biases were related, that is, spider-phobic females overestimated the risk that spiders were present in a room and also exaggerated the probability of negative consequences of such an encounter. Dijk and de Jong (2009) argue that these two biases do not tap into the same cognitive anticipatory process. In their study, they found evidence for this assumption and showed a divergence between these biases. According to their findings, individuals with blushing-phobia tend to overestimate the likelihood that they blush while they do not report excessive concern about the negative outcomes of blushing. So far, these findings reveal an inconsistent pattern and a gap in the research which (a) systematically investigates these two biases together and (b) draws a clear line between these two kinds of biased risk perceptions.

Indeed, few studies in the covariation bias literature assessed base rates for fear-relevant stimuli which might be seen as a form of an encounter bias (e.g., Pauli et al., 1998; Garner et al., 2006). None of these studies found evidence for heightened risk estimates related to fear-relevant encounters in fearful individuals. Instead, all individuals seem to have higher estimated risk of such encounters. This finding might indicate that risk estimates of negative encounters are separate to risk estimates of negative outcomes following fear-relevant encounters.

A further consideration addresses the specificity of the encounter of fear-relevant events and their negative outcomes. So far, research has lacked systematical investigation into whether biased risk perception is specific to an individual’s fear or generalized to all negative events. To our knowledge, there are only two studies that have tried to address this question. An investigation with claustrophobics indicated that participants only overestimate the risks specific to their fear, showing evidence of a domain-specific overestimation (Öst and Csatlos, 2000). There is also evidence for domain-specific overestimations for the domains of fear of flying, heights, and public speaking (Gursky and Reiss, 1987).

In summary, it has been convincingly argued that fear is conceptually related to risk perceptions. Specifically, fearful individuals overestimate the risk of negative events. Laboratory studies have demonstrated convincingly that fearful individuals tend to overestimate the occurrence of aversive outcomes in the presence of fear-related stimuli. However, there is a lack of research that systematically differentiates between encounters with the fear-relevant objects or situations on the one hand, and the negative outcomes of such encounters on the other. Moreover, only a few studies have questioned fearful individuals about the probability of fear-related encounters that can occur in real life and their negative impact.

Furthermore, the specificity of these two biases for specific fear-relevant material (see Berdica et al., 2018) warrants further examination. Thus, the two types of biases need to be studied in a single design with more than one fear domain.

We set out to investigate the relationship between the two biases and to find out if overestimations of encounters and negative outcomes of such encounters are specific to a given fear domain, i.e., fear of spiders, snakes, or everyday fear triggers. To this end, we developed two ecologically valid risk questionnaires for an online-survey: One relating to potential encounters with fear-relevant situations and another that focuses on the aversive outcomes of such encounters. Concerning the ecological validity, we picked three fear domains that are common in clinical samples (see Hofmann et al., 2008). Two domains of animal fears (fear of spiders and fear of snakes) were selected because they were comparable in terms of their evolutionary basis and prevalent in clinical samples. While these were, thus, similar but distinct fear domains they were contrasted with a third domain of fears elicited by situations typically relating to generalized worries (see Tallis and de Silva, 1992; Cartwright-Hatton and Wells, 1997). We recruited a large sample of participants varying in their degree of fear to provide for a sufficient number of high fearful and low fearful individuals on these fear domains. We expected that domain-specific estimation of risk vary as a function of the specific fears in each one of the related domains. Overall, we expected higher risk ratings in more fearful individuals.

## Materials and Methods

### Participants

Participants from various universities in German speaking countries from different departments of Psychology were recruited via e-mail lists. Two psychology journals advertised the survey on their homepages and social media platforms. For our analyses, the remaining dataset of 630 people was used (*M* = 30.11, *SD* = 10.30, Range = 16–66, 76.7% females). For extreme group differences, we also assessed the degree of spider and snake fearfulness as well as the degree of worry tendencies. According to these prior studies and recommendations (see section below), 320 participants were classified as low in fear of spiders and 221 as high spider fearfuls. For the fear of snakes, 473 were classified as low in fear of snakes and 39 as high snake fearfuls. In terms of worry tendencies, 101 were classed as high worriers and 529 as low worriers. Questionnaire scores and demographic data are presented in Table 1.

**TABLE 1:** Demographics and questionnaire data.

	**High spider fearful**	**Low spider fearful**	***t/χ^2^***	***p*-values**
	
***n***	**221**	**320**		
Age	27.28 (8.31)	32.26 (11.14)	−5.95^a^	<0.001
Sex = Female	199 (90.0%)	212 (66.3%)	42.41^b^	<0.001
Depression (BDI)	33.23 (10.64)	29.36 (8.38)	4.53^a^	<0.001
State anxiety (STAI-S)	42.01 (11.99)	37.88 (11.03)	4.08^a^	<0.001
Trait anxiety (STAI-T)	44.46 (12.44)	38.72 (11.54)	5.50^a^	<0.001
Optimism (LOT-R)	21.48 (4.65)	23.16 (4.54)	−4.21^a^	<0.001
Fear of spiders (FSQ)	44.90 (22.72)	1.39 (1.94)	28.40^a^	<0.001
Fear of snakes (SNAQ)	9.10 (6.75)	5.30 (4.60)	7.28^a^	<0.001
Worry tendencies (PSWQ)	50.33 (13.65)	42.65 (12.62)	6.73^a^	<0.001

	**High snake fearful**	**Low snake fearful**	***t/χ^2^***	***p***
	
***n***	**39**	**473**		

Age	27.59 (8.25)	30.39 (10.03)	1.70^a^	0.090
Sex = Female	348 (73.6%)	34 (87.2%)	10.20^b^	0.006
Depression (BDI)	32.72 (12.04)	30.56 (9.34)	1.35^a^	0.176
State anxiety (STAI-S)	41.46 (12.97)	38.93 (11.46)	1.31^a^	0.190
Trait anxiety (STAI-T)	42.18 (12.43)	40.48 (12.11)	0.94^a^	0.401
Optimism (LOT-R)	22.62 (4.55)	22.59 (4.63)	0.03^a^	0.978
Fear of spiders (FSQ)	30.18 (27.57)	15.06 (23.30)	3.33^a^	0.002
Fear of snakes (SNAQ)	23.38 (2.85)	4.12 (2.35)	41.03^a^	<0.001
Worry tendencies (PSWQ)	48.97 (14.22)	45.00 (13.35)	1.78^a^	0.076

	**High worry**	**Low worry**	***t/χ^2^***	***p*-values**
	
***n***	**101**	**529**		

Age	27.62 (8.57)	30.59 (10.54)	3.06^a^	0.003
Sex = Female	140 (88.6%)	343 (72.7%)	8.82^b^	0.003
Depression (BDI)	42.51 (12.37)	28.94 (7.34)	12.68^a^	<0.001
State anxiety (STAI-S)	52.36 (11.46)	37.43 (10.18)	12.20^a^	<0.001
Trait anxiety (STAI-T)	58.40 (8.83)	38.15 (9.87)	19.21^a^	<0.001
Optimism (LOT-R)	17.31 (3.71)	23.33 (4.14)	13.62^a^	<0.001
Fear of spiders (FSQ)	28.58 (30.57)	15.82 (22.27)	4.00^a^	<0.001
Fear of snakes (SNAQ)	8.41 (6.74)	6.60 (3.98)	1.59^a^	0.134
Worry tendencies (PSWQ)	67.63 (4.39)	42.13 (10.31)	24.24^a^	<0.001

*Means and standard deviations separately for high and low spider fearful, for high and low snake fearful participants as well as for high worriers and low worriers. n, number of participants; BDI, Beck Depression Inventory (Hautzinger et al., 2006); STAI-S and -T, State and trait version of the State-Trait Anxiety Inventory (Laux et al., 1981); LOT-R, revision of the Life-Orientation-Test (Glaesmer et al., 2008); FSQ, Fear of Spiders Questionnaire (Rinck et al., 2002); SNAQ, Snake Questionnaire (Klorman et al., 1974); PSWQ, Penn State Worry Questionnaire (Meyer et al., 1990); ^*a*^*t* score for group comparison. ^*b*^*χ^2^* score for gender ration comparison.*

#### Dropout and Case Exclusion

Initially, 1039 individuals started the online survey; however, only 659 (37% dropout) of them completed all questions. This dropout rate is comparable to the average drop-out rates if other online-surveys (e.g., Galesic, 2006).

To provide a high data quality, participants were asked to provide more information on three items to help us understand the plausibility of their responses at the end of the survey. One question asked for the degree of accuracy of one’s answer on a scale from 1 to 4 (“I do not agree” to “I agree”). In addition, the other two questions asked if participants answered the questionnaires honestly and if there were any reasons not to use their data. We excluded all participants who did not answer the plausibility questions and who were not debriefed due to early termination as participants were provide with the final and more detailed debriefing at the end of the survey. Participants were also excluded if they either (a) rated the accuracy question with “I rather disagree” or (b) if a participant admitted to dishonest answers or (c) if they answered with “Yes” for any reasons not to use their data. Therefore, we excluded 29 participants due to a questionable data quality.

### Procedure and Materials

#### Control Measures

Participants were granted access to the survey platform *soscisurvey* via an online link. First, they were provided with general information on the questionnaires and personal data to be assessed. After giving informed consent, participants filled in a questionnaire battery. They provided demographic information and then gave information about their current symptoms of depression with the Beck Depression Inventory (BDI-II; Beck et al., 1996; German version: Hautzinger et al., 2006). This questionnaire consists of 21 items measuring different aspects of depressive symptoms (e.g., sadness, sleep disorder, and fatigue) on a 0–4 scale with higher values indicating more severe symptoms of depression. State and trait anxiety were assessed with the State-Trait-Anxiety Inventory (STAI; Spielberger et al., 1983; German version: Laux et al., 1981). In this inventory, state anxiety and trait anxiety were measured with 20 items each; participants indicated scores on a 1–4 Likert-type scale (“Not at all” to “Totally”). Optimism was assessed with the Life orientation test (LOT-R; Scheier and Carver, 1985; German version: Glaesmer et al., 2008) that consists of ten items rated on a 1–4 likert-type scale (“Does not apply at all” to “Totally applies to me”).

For all questionnaires, sum scores were curated for each participant and averaged across the sample for the statistical analyses.

#### Fear Domains

Levels of fear of spiders were assessed with the Fear of Spider Questionnaire (FSQ; Rinck et al., 2002), which is a 18-item questionnaire with an answer format from 0 to 6 (“Does not apply at all” to “Totally applies to me”). It shows high reliability and validity as well as high specificity and good sensitivity in differentiating fearful and non-fearful individuals. According to recommendations of Rinck et al. (2002) and previously published articles with the same cut-offs (e.g., Pittig et al., 2018), participants with a score of 6 or lower were classified as low spider fearful participants and those with a score of 15 or higher as high spider fearful participants.

We assessed the degree of snake fear with the Snake anxiety Questionnaire (SNAQ; Miltner et al., 2005). The SNAQ is a questionnaire that captures current symptoms of fear of snakes with 30 dichotomous items. Following the recommendations of prior studies with the Snake Questionnaire (SNAQ; Klorman et al., 1974; Miltner et al., 2005), a score lower than 9 indicates low snake fearfulness and a score higher than 20 indicates high snake fearfulness.

Worry tendencies were measured using the Penn State Worry Questionnaire (PSWQ; Meyer et al., 1990), which is widely used and captures symptoms of generalized anxiety disorder with 16 items on a 1–5 scale (“Not typical for at all” to “Totally typical for me”). We used a cut-off of 62 or greater to assign people to the high worrier group and a score of 61 or lower to assign people to the low worrier group. A cutoff of 62 for the PSWQ is a recommended used score for maximizing the specificity and negative predictive power in a clinical and analog samples for a diagnosis of generalized anxiety disorders (Behar et al., 2003). Risk estimations for fear-relevant encounters were assessed with a newly developed 23-item questionnaire and negative outcomes of such encounters with a 21-item questionnaire.

Finally, participants answered the three plausibility questions and were debriefed and informed about the survey topic. In the end, all of them had the opportunity to specify their e-mail address to take part in a lottery as a gratification for their participation. If they agreed, they took part in the lottery and three of them had the chance to win a voucher for an online shopping portal.

### Risk Estimations

To disentangle the different components of risk estimation, we developed two questionnaires. One, assessing probability estimations of specific and general risks for encountering an aversive event (*Risk of encounter questionnaire*, REQ), and another one that addresses the expected negative outcomes of these events (*Risk of negative outcome questionnaire*, RNOQ).

For more details on item selection strategies as well as validity and reliability analyses please refer to Supplementary Analyses (see section “Confirmation of the Data Structures”).

#### Fear-Relevant Encounters

The REQ is a 21-item questionnaire that captures three different *Encounter Domain*, “spider,” “snake” and “everyday fear triggers,” respectively. The *Encounter Domain* “spider” and “snake” include seven items each consisting of locations where these types of animals may be found (e.g., forest, cellars etc.). The wording of these items was the same except for the name of the respective animal (spiders vs. snakes). So, for each spider-item there was a parallel snake-item. For the worry-related events, we referred to the content of common items of questionnaires measuring components and the severity of Generalized Anxiety Disorders (GAD) (e.g., Worry Domain Questionnaire; Tallis et al., 1992; Meta-Cognition-Questionnaire; Möbius and Hoyer, 2003). We also referred to expert opinions of five clinical psychologists and three psychotherapists working at our department.

We created to answer scales to specify the time period the items of the REQ refer to. One scale refers to the period in the course of the next 12 months and the other one of the period in the course of your life. As the probability estimations for the two different time periods were highly correlated (Range: 0.52–0.88) per item, we built a sum score of the ratings on the two scales referring to the two different time periods for each item. This was in order to have an overall score for the probability estimations of the event not depending on the time period they referred to.

The resulting 23 items (seven for the *Encounter Domain* “spider,” and eight for the domains of “snake” and “everyday fear triggers,” respectively) were rated on a 1–7 Likert-scale (“very unlikely” to “very likely”). This scaling was used based on a questionnaire developed by Nesse and Klaas (1994) which assessed the risk perceptions of patients with anxiety disorders.

#### Negative Outcomes of Fear-Relevant Encounters

For risk estimation of negative outcomes following fear-relevant encounters, we developed the RNOQ with 20 items that were assigned to four different *Outcome Domains*, “danger-based fear,” “anxiety-based fear of spiders,” “anxiety-based fear of snakes,” and “general catastrophizing,” according to the results of the confirmatory factor analysis (see Supplementary Analyses). For the *Outcome domain* “danger-based anxiety” we combined two spider- and four snake-items, because they show high loadings on the same factor and the whole scale showed sufficient reliability on measures (see Supplementary Analyses). Consequently, this scale consisted of six items, two addressing outcomes relating to bodily harm when encountering a spider (e.g., being bitten by a spider), and four addressing bodily harm resulting from a snake encounter. The two *Outcome Domains* “anxiety-based fear of spiders,” “anxiety-based fear of snakes” include three items each referring to panic symptoms when a fear-relevant object is encountered. Prior findings (Gursky and Reiss, 1987) also confirmed a differentiation of danger-based and anxiety-based fears when being confronted with fear-relevant events. The last *Outcome Domain*, “generalized catastrophizing,” includes eight items and assesses the risk of catastrophizing when being confronted with everyday fear triggers.

All 20 items were again rated using 21 items on a 1–7 Likert-scale (“very unlikely” to “very likely”) based on previous studies (Nesse and Klaas, 1994).

### Statistical Analysis

Statistical analyses were run by SPSS 24 (SPSS Inc., 2016) and R 3.4.3 (R Core Team, 2017). We conducted correlation and regression analyses to calculate if the degree of specific fears has incremental predictive value over the other measures, which are not known to strongly influence these risk estimations. We therefore added predictors in the regression models that are significantly related to the sum scores of the subscales of the two risk questionnaires.

To test for specific encounter as well as outcome biases in high fearful individuals, we conducted separate mixed analyses of variance. This included factors *Encounter Domain* for the REQ and *Outcome Domain* for the RNOQ as within-factors, and *Fear/Worry* as between subject factor. For between-group factors, we built extreme groups of high and low fearful individuals regarding the specific fear domains (spider, snake, and everyday fear triggers). Finally, group differences regarding the fear domain as well as the *Encounter* and *Outcome Domains* of the two questionnaires were examined. We decided to not correct for multiple comparisons. Even if we had done so, results would remain the same.

In order to interpret correlations, we follow conventions of *r* = 0.10 for a small, *r* = 0.30 for a medium and *r* = 0.50 for a large effect size (Cohen, 1988).

## Results

### Domain- Specificity of the Risk Estimations of Encounter Domains

To examine the factors that might be predictive for fear-relevant encounters and their negative outcomes, we first run correlational analyses between the *Encounter* and *Outcome Domains* and all other measures (see Table 2). In a second step, only predictors that showed a significant relationship to the different domains were entered in a stepwise regression.

**TABLE 2:** Correlation analyses between the specific fears and risk estimations.

		***N***	**Mean (SD)**	**1**	**2**	**3**	**4**	**5**	**6**	**7**	**8**	**9**	**10**	**11**	**12**
1	Fear of spiders (FSQ)	630	17.87 (24.23)	1											
2	Fear of snakes (SNAQ)	630	7.06 (6.04)	0.20^∗∗^	1										
3	Worry tendencies (PSWQ)	630	46.22 (13.42)	0.27^∗∗^	0.18^∗∗^	1									
4	REQ (total)	630	167.67 (32.11)	0.04	−0.02	0.12^∗∗^	1								
5	REQ (spider encounter)	630	76.60 (16.26)	0.04	−0.12^∗∗^	−0.01	0.77^∗∗^	1							
6	REQ (snake encounter)	630	31.52 (12.76)	−0.02	0.07	0.01	0.63^∗∗^	0.22^∗∗^	1						
7	REQ (encounter of everyday fear triggers)	630	59.56 (15.15)	0.07	0.04^∗∗^	0.26^∗∗^	0.76^∗∗^	0.37^∗∗^	0.27^∗∗^	1					
8	RNOQ (total)	630	46.29 (15.91)	0.54^∗∗^	0.51^∗∗^	0.45^∗∗^	0.25^∗∗^	0.06	0.19^∗∗^	0.32^∗∗^	1				
9	RNOQ (danger-based fear)	630	8.98 (4.22)	0.29^∗∗^	0.50^∗∗^	0.19^∗∗^	0.18^∗∗^	0.01	0.27^∗∗^	0.16^∗∗^	0.73^∗∗^	1			
10	RNOQ (anxiety-based spider fear)	630	7.94 (5.08)	0.84^∗∗^	0.24^∗∗^	0.31^∗∗^	0.10^*^	0.04	0.02	0.14^∗∗^	0.69^∗∗^	0.32^∗∗^	1		
11	RNOQ (anxiety-related snake fear)	630	8.01 (4.89)	0.23^∗∗^	0.70^∗∗^	0.22^∗∗^	0.15^∗∗^	0.05	0.07	0.20^∗∗^	0.73^∗∗^	0.55^∗∗^	0.38^∗∗^	1	
12	RNOQ (generalized catastrophizing)	630	21.36 (7.29)	0.26^∗∗^	0.19^∗∗^	0.51^∗∗^	0.29^∗∗^	0.08	0.19^∗∗^	0.38^∗∗^	0.79^∗∗^	0.42^∗∗^	0.36^∗∗^	0.35^∗∗^	1

^*^Correlation is significant at the 0.05 level (2-tailed) and ^∗∗^correlation is significant at the 0.01 level (2-tailed). The shades of gray indicate the intra-correlations between the different fear domains, encounter, and outcome domains (e.g., danger-based anxiety with generalized catastrophizing); *N*, number of participants; FSQ, Fear of Spiders Questionnaire (Rinck et al., 2002); SNAQ, Snake Questionnaire (Klorman et al., 1974); PSWQ, Penn State Worry Questionnaire (Meyer et al., 1990); REQ, Risk encounter questionnaire; RNOQ, Risk of negative outcomes questionnaire.

#### Spider and Snake Encounters

The relationship between the *Encounter Domain* “Spider” and the degree of specific fears only revealed a significant and negative relationship with fear of snakes, *r* = −0.12, *p* < 0.001, and with no other measures, all *r*s ≤ 0.05, all *p*s ≥ 0.102. Therefore, a stepwise regression was obsolete.

Estimations of the risk to encounter a snake were correlated with depression, *r* = 0.09, *p* = 0.034, and optimism, *r* = −0.09, *p* = 0.022. In an ensuing stepwise regression, only optimism accounted for a significant amount of explained variance, β = −0.25, *t*(629) = −2.29, *p* = 0.022; model fit: *F*(1,628) = 5.25, *p* = 0.022, *corrected R^2^* = 0.01.

#### Everyday Fear Triggers

Risk estimates to encounter everyday fear triggers were significantly related to state, *r* = 0.26, *p* < 0.001, as well as to trait anxiety, *r* = 0.29, *p* < 0.001. Furthermore, the relationships between this *Encounter Domain* and depression, worry tendencies and optimism proved significant, *r* = 0.23, *p* < 0.001, *r* = 0.26, *p* < 0.001, *r* = −0.21, *p* < 0.001. In the following stepwise regression, only trait anxiety explained a significant amount of the variance in the data, β = 0.36, *t*(629) = 7.57, *p* < 0.001, and showing incremental prediction over the other measures, β ≤ 0.09, *t*(629) ≤ 1.36, *p* ≥ 0.176; model fit: *F*(1,628) = 57.37, *p* < 0.001, *corrected R^2^* = 0.08.

In sum, the exploratory analyses of REQ did not confirm a domain-specific risk overestimation of fear-relevant encounters in fearful individuals. The final models of the regression analyses are shown in Table 3.

**TABLE 3:** Summary of the steps of the stepwise regression analyses for the variables predicting risk estimations of the REQ and RNOQ (*N* = 630).

**Scales of the REQ/RNOQ and step**	**Predictor**	***R***	***R*^2^-Change**	***p*-value**
Snake encounter				
1	LOT-R	0.09	0.01	0.022
Encounter everyday fear triggers				
1	STAI-T	0.29	0.08	<0.001
Danger-based fear				
1	SNAQ	0.50	0.25	<0.001
2	FSQ	0.54	0.04	<0.001
3	LOT-R	0.55	0.02	<0.001
Anxiety-based spider fear				
1	FSQ	0.84	0.71	<0.001
2	PSWQ	0.85	0.01	<0.001
3	SNAQ	0.85	0.00	0.004
Anxiety-based snake fear				
1	SNAQ	0.70	0.49	<0.001
2	PSWQ	0.70	0.01	0.001
3	FSQ	0.71	0.00	0.025
Generalized catastrophizing				
1	PSWQ	0.51	0.26	<0.001
2	LOT-R	0.56	0.05	<0.001
3	FSQ	0.57	0.02	<0.001
4	BDI-II	0.58	0.01	0.002
5	SNAQ	0.59	0.01	0.005

*BDI, Beck Depression Inventory (Beck et al., 1996); STAI -T, Trait version of the State-Trait Anxiety Inventory (Laux et al., 1981); LOT-R, revision of the Life-Orientation-Test (Glaesmer et al., 2008); FSQ, Fear of Spiders Questionnaire (Rinck et al., 2002); SNAQ, Snake Questionnaire (Klorman et al., 1974); PSWQ, Penn State Worry Questionnaire (Meyer et al., 1990).*

### Domain Specificity of the Risk Estimations of Outcome Domains

For the risk of negative outcomes, all *Outcome Domains* revealed significant relationships to all additional measures, all *r*s ≥ 0.11, all *p*s < 0.001.

#### Danger-Based Fear

For the *Outcome Domain* “danger-based fear,” only fear of snakes, β = 0.32, *t*(629) = 13.39, *p* < 0.001, and spiders, β = 0.03, *t*(629) = 5.14, *p* < 0.001, as well as optimism, β = −0.11, *t*(629) = −3.63, *p* < 0.001, were significant predictors of the probability estimations. Fear of snakes was the most powerful predictor which was entered first into the model: *F*(3,626) = 91.33, *p* < 0.001, *corrected R^2^* = 0.30.

#### Anxiety-Based Spider and Snake Fear

The estimations of anxiety-based fear by encountering a spider were significantly predicted by fear of spiders as the measure with the most predictive power, β = 0.17, *t*(629) = 36.41, *p* < 0.001, followed by worry tendencies, β = 0.03, *t*(629) = 3.38, *p* ≤ 0.001, and fear of snakes, β = 0.05, *t*(629) = 2.89, *p* = 0.004, and accounted for the best model fit: *F*(3,626) = 539.44, *p* < 0.001, *corrected R^2^* = 0.72.

For “anxiety-based snake fear,” predictors were the same; however, with a different order: fear of snakes, β = 0.54, *t*(629) = 23.01, *p* < 0.001, worry tendencies, β = 0.03, *t*(629) = 2.82, *p* = 0.005, and fear of spiders, β = 0.01, *t*(629) = 2.25, *p* = 0.025; model fit: *F*(3,626) = 208.80, *p* < 0.001, *corrected R^2^* = 0.71.

#### Generalized Catastrophizing

The final model with the best fit for the estimations of the *Outcome Domain* “generalized catastrophizing,” model fit: *F*(5,624) = 65.67, *p* < 0.001, *corrected R^2^* = 0.59, was the model with the best predictor of worry tendencies, β = 0.13, *t*(629) = 5.63, *p* < 0.001, followed by optimism, β = −0.37, *t*(629) = −5.14, *p* < 0.001, and fear of spiders, β = 0.03, *t*(629) = 3.09, *p* = 0.002, depression scores, β = 0.10, *t*(629) = 3.11, *p* = 0.002, and finally fear of snakes, β = 0.11, *t*(629) = 2.79, *p* = 0.005. Table 3 shows a summary of the explained variances in the different models and the changes by entering incremental predictors in each step of the regression analyses.

According to the predictive power of the separate measures, these findings revealed that probability estimations of negative outcomes can be best predicted by fears which are domain specific to the outcome they referred to. This pattern indicated a kind of domain specificity for the probability estimations of the outcomes of fear-relevant encounters compared to the estimations of the encounters themselves.

**FIGURE 1:**
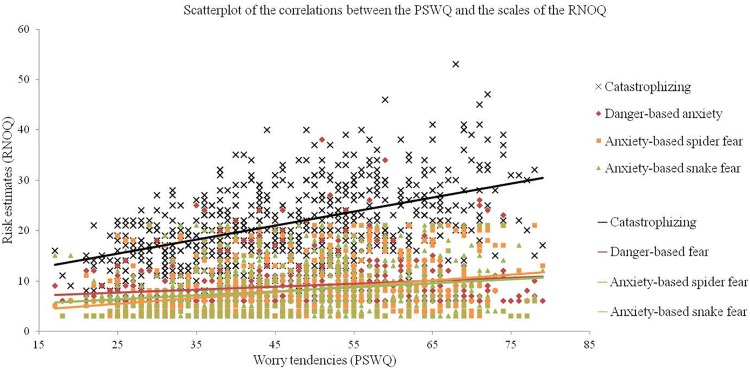
Relationships between worry tendencies (PSWQ) and risk estimates (scales of the RNOQ). The lines mark the linear trend of the relationships.

To illustrate, the relationships between worry tendencies and the different scales of the RNOQ are shown in Figure 1. Additional correlational data and figures of the relationships of the other measures of fear domains and the RNOQ-scales are presented in Supplementary Materials.

### Extreme Group Differences in Risk Estimations of Fear-Relevant Encounters

#### High Spider Fearful vs. Low Spider Fearful Individuals

A repeated measure ANOVA with *Encounter Domain* (spider vs. snake vs. everyday fear triggers) as within and *Fear of spiders* (yes vs. no) as between factor only revealed a significant main effect of the *Encounter Domain*, *F*(2,1078) = 1707.98, *p* < 0.001, $\eta_{p}^{2}$ = 0.76. *Fear of spiders* and the interaction between the two factors did not reach significance, all *F*s ≤ 1.32, all *p*s ≥ 0.268, all $\eta_{p}^{2}$s *≤* 0.01.

#### High Snake Fearful vs. Low Snake Fearful Individuals

The ANOVA with the between group factor *Fear of snakes* revealed the same results as the fear domain of spiders: a significant main effect of the *Encounter Domain*, *F*(2,1020) = 105.62, *p* < 0.001, $\eta_{p}^{2}$ = 0.50, and no significant main effect of *Fear of snakes* and interaction, all *F*s ≤ 1.60, all *p*s ≥ 0.203, all $\eta_{p}^{2}$s *<* 0.00.

All participants rated the risk of encountering spiders higher than encountering snakes, *t*(540) = 57.38, *p* < 0.001, *d* = 4.94, or encountering everyday fear triggers, *t*(540) = 23.15, *p* < 0.001, *d* = 1.99, and the risk of encountering everyday fear triggers more likely than encountering a snake, *t*(540) = 36.96, *p* < 0.001, *d* = 3.18. This pattern was the same for high snake fearfuls and low fearfuls, all *t*s ≥ 23.06, all *p*s < 0.001, all *d*s ≥ 1.98.

#### High vs. Low Worriers

For the extreme groups of high and low worriers, there was a significant main effect of the *Encounter Domain, F*(1.97, 1242.29) = 1178.82, *p* < 0.001, $\eta_{p}^{2}$ = 0.65, and a significant interaction of the *Encounter Domain* and *Worry, F*(1.97, 1242.29) = 12.36, *p* < 0.001, $\eta_{p}^{2}$ = 0.02. However, the main effect of *Worry* revealed no significance, *F*(1,628) = 2.48, *p* = 0.116, $\eta_{p}^{2}$ = 0.00. Both groups rated the risk of encountering a spider more likely than risk of encountering a snake, *t*(629) = 61.71, *p* < 0.001, *d* = 4.92, and the risk of encountering everyday fear triggers, *t*(629) = 24.16, *p* < 0.001, *d* = 1.93. They were also more likely to perceive higher risk of encountering everyday fear triggers than the risk of encountering a snake, *t*(629) = 41.32, *p* < 0.001, *d* = 3.30.

High worriers compared to low worriers did not differ in their risk estimates of encountering a spider, *t*(628) = 0.37, *p* = 0.708, *d* = 0.03, or a snake, *t*(628) = 0.82, *p* = 0.410, *d* = 0.07. The two groups only differed in their risk estimates of encountering everyday fear triggers, *t*(628) = 4.50, *p* < 0.001, *d* = 0.36. Thus, high worriers only had higher risk estimates in their feared domain of encountering everyday fear triggers.

In sum, anxious participants had the same perception of the risk to encounter fear-relevant stimuli as non-anxious ones. Iinterestingly, high worriers had an overestimation to encounter fear-relevant events compared to low worriers. This was especially prevalent in general and domain-specific overestimations of the risk to encounter everyday fear triggers.

### Extreme Group Differences in Risk Estimations of Negative Outcomes

We conducted mixed ANOVAs separately for the extreme groups for each fear domain (spiders/snakes/worriers) as between-subjects factor and *Outcome Domain* (Danger-based vs. Anxiety-based spider fear vs. Anxiety-based snake fear vs. Generalized catastrophizing) as within-subjects factor.

**FIGURE 2:**
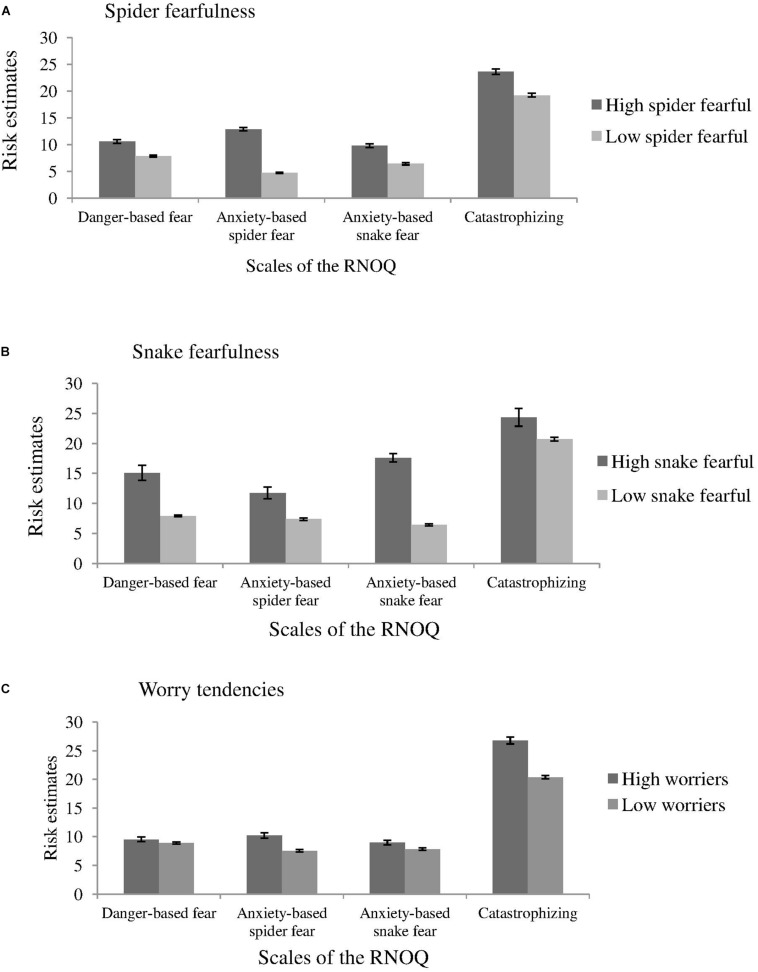
Mean risk estimates of the extreme groups of each fear domain on the scales of the RNOQ. Error bars reflect the standard error of means. **(A)** Fear domain of spiders. **(B)** Fear domain of snakes. **(C)** Fear domain of everyday fear-triggers.

There were significant main effects of *Outcome Domain* for the extreme groups of each fear domain, all *F*s ≥ 288.12, all *p*s < 0.001, all $\eta_{p}^{2}$s *≥* 0.36, as well as significant main effects for the extreme groups of each fear domain, all *F*s ≥ 77.88, all *p*s < 0.001, all $\eta_{p}^{2}$s *≥* 0.11, and for each of their interactions, all *F*s ≥ 24.54, all *p*s < 0.001, all $\eta_{p}^{2}$s *≥* 0.05 (see Figure 2).

Overall, the risk of negative outcomes of everyday fear triggers was higher than all other estimates of negative outcomes across all extreme groups, all *t*s ≥ 5.01, all *p*s < 0.001, all *d*s ≥ 1.63. High fearful individuals rated the total risk of negative outcomes by encountering fear-relevant events as higher than their low fearful counterparts, all *t*s ≥ 7.51, all *p*s < 0.001, all *d*s ≥ 2.37.

#### High Spider Fearful vs. Low Spider Fearful Individuals

High spider fearfuls compared to low spider fearfuls had higher risk estimates on all scales of the RNOQ, all *t*s ≥ 7.28, all *p*s < 0.001, all *d*s ≥ 0.70. Furthermore, high spider fearful individuals overrated the risk of anxiety-based fear from encountering a spider compared to the anxiety-based fear from encountering a snake, *t*(221) = 6.76, *p* < 0.001, *d* = 0.91, and compared to danger-based fear, *t*(221) = 5.06, *p* < 0.001, *d* = 0.68. On the contrary, low spider fearfuls had lower risk estimates of anxiety-based spider fear compared to all other scales, all *t*s ≥ 8.76, all *p*s < 0.001, all *d*s ≥ 0.98.

#### High Snake vs. Low Snake Fearful Individuals

High compared to low snake fearful individuals gave higher risk estimates on all scales of the RNOQ, all *t*s ≥ 2.39, all *p*s ≤ 0.021, all *d*s ≥ 0.74. For high snake fearful individuals, risk estimates for anxiety-based fear by snakes were higher than estimates for anxiety-based spider fear, *t*(38) = 6.03, *p* < 0.001, *d* = 1.96, and higher than estimates of danger-based fear, *t*(38) = 2.22, *p* < 0.001, *d* = 0.72. For individuals who were low in snake fear, this pattern was in the opposite direction: risk estimates of anxiety-based snake fear were lower compared to all other estimates of negative outcomes, all *t*s ≥ 4.09, all *p*s < 0.001, all *d*s ≥ 0.38.

#### High vs. Low Worriers

The corresponding pattern was found for high worriers and low worriers: High worriers compared to low worriers exaggerated all negative outcomes, all *t*s ≥ 2.13, all *p*s ≤ 0.03, all *d*s ≥ 0.17, with the exception of danger-based anxiety by encountering spiders or snakes, *t*(628) = 1.40, *p* = 0.163, *d* = 0.11. Especially for high worriers, risk estimates of negative outcomes by encountering everyday fear triggers were higher than risk estimates on all other scales of the RNOQ, all *t*s ≥ 17.89, all *p*s < 0.001, all *d*s ≥ 3.58. This pattern was almost the same for low worriers, all *t*s ≥ 42.91, all *p*s < 0.001, all *d*s ≥ 3.72. However, low worriers also rated the risk of danger-based fear higher than the anxiety-based fear by a spider, *t*(528) = 5.95, *p* < 0.001, *d* = 0.52, or a snake encounter, *t*(528) = 5.48, *p* < 0.001, *d* = 0.48. Thus, high worriers showed a more specific overestimation in their feared domain than did low worriers.

To conclude, high fearful individuals generally overestimated risk of all negative outcomes. However, the significant interaction revealed that this overestimation is mostly pronounced in the feared domain.

## Discussion

Dangerous situations generally evoke fear, but the specific relationship between fear and risk estimations has not been well understood. This is the first study to systematically differentiate risk estimates of fear-relevant encounters and the negative outcomes of such encounters. Our findings emphasize the idea that these concepts should be studied separately. In highly fearful individuals, risk assessments were exaggerated with respect to the *outcomes* of all negative encounters but most pronounced in fear-relevant encounters. The results provide further evidence that risk perception biases could be assessed in a domain-specific manner and are specific to an individual’s fear domain, particularly so with respect to negative outcomes.

Previous research has primarily investigated biased risk estimations of negative outcomes (see: Wiemer and Pauli, 2016). We did not find any studies that systematically differentiated whether biased risk perception is related to negative outcomes or rather to fear-relevant encounters. There was also none that related these two kinds of risk perception. However, our data shows that it is worthwhile to separate these two types of risk estimations and focus also on encounters of fear-relevant events. In our data these two concepts are not highly correlated and show relationships with other specific fear measures. The possibility that poor reliability might be the reason for lower correlations is not likely – all scales had sufficient to high reliability scores (see Supplementary Materials).

In line with other recent findings (Aue and Hoeppli, 2012), we did not find heightened risk estimates of fear-relevant encounters in high fearful compared to low fearful individuals. However, Aue and Hoeppli (2012) found an encounter bias within high spider fearfuls as they rated the risk of encountering their fear-relevant stimuli higher than the risk of encountering other stimuli. These conflicting findings might be due to the different methodical approaches; the authors used a more artificial and complex paradigm whereas we focused more on an ecologically valid method with a huge sample. Thus, future research should investigate biased risk estimations in high fearful individuals within a multi-methodical approach by combining different measures of risk estimations.

In the few studies that exist, there are also conflicting findings. The specificity of the risk estimates between the encounter of fear-relevant events and their negative outcomes was also found by Dijk and de Jong (2009), but in the opposite way: In their study, individuals who have a strong fear of blushing overestimated the risk of blushing. However, their ratings of the negative effects of blushing were not different than their fearful and non-fearful counterparts. However, blushing may be a very special fear because it can also be considered the negative outcome of an embarrassing social event (the encounter).

Interestingly, only high worriers rated the risk of fear-relevant events in their fear-relevant domain more likely than low worriers. One reason could be the wide range of situations of the domain because a wide range of fear-relevant events were rated rather than only the encounter of a specific stimulus. Research should therefore take a larger variety of fears into account to investigate types of biased risk perception in more fear domains.

For risk estimations of *negative outcomes*, high fearful individuals showed a generalized risk overestimation of all sorts of negative outcomes independently of their fear domain. Importantly, results indicate that the relationship between risk overestimations of negative outcomes was most pronounced in an individual’s specific fear domain. Moreover, risk overestimations of negative outcomes of fear-relevant encounters could be best predicted by the specific fear domain. These findings provide further insight into a domain-specific risk perception of negative outcomes which extended previous literature that only assessed either one fear domain or only uses one fear-relevant material (e.g., Lovibond et al., 1994; Diamond et al., 1995; de Jong et al., 1998; van Overveld et al., 2010; Duits et al., 2016). Results of a domain-specific overestimation were also confirmed by regression analyses; the degree of fear in the relating domain had the most predictive power for the domains of risk estimations of negative outcomes.

Furthermore, results show that danger and anxiety risk estimations load on separate factors. Anxiety-based risk estimates showed an even more domain-specific structure as it could be differentiated between anxiety-based risk expectancies for spiders as well as for snakes. These findings are in line with prior findings that indicate a danger and anxiety-based structure of risk estimations (Gursky and Reiss, 1987). This brings to attention the further need to investigate the particular content of risk estimations systematically, even if there is still a lack of studies in this area. Additional research is needed to further confirm the data structure with clinical samples, for example participants with specific phobias across different fear domains or generalized anxiety disorder.

Interestingly, high spider and high snake fearful individuals especially overestimated their risk of panicking when encountering their specific feared animal and being harmed by these animals. Most studies only examined the perceived association between a fear-relevant stimulus and an aversive outcome such as an electric shock (e.g., in the covariation bias paradigm). The latter seems to be more similar to harm than the experience of panic. Therefore, future research should involve an experimental design that covers both kinds of negative outcomes, danger-based and anxiety-based.

Our results must be interpreted with due caution. First, they are based on an online-survey which provide less control as could be achieved in a laboratory. However, recent research has documented that paper-pencil and computer-based (Gwaltney et al., 2008) or Internet-based assessments (Weigold et al., 2013) yield equivalent self-report.

What is more, high fearful and low fearful individuals were identified post-hoc. Thus, samples differed in sizes and other important variables, e.g., gender, that may play as confounding variables. We used *Levene*’s-test to account for the difference in sample size which might have resulted in less statistical power (Rusticus and Lovato, 2014). However, because our assessment was reliable and our samples large, there was enough power to test our hypotheses.

In addition, our sample only consisted of high and low fearful participants and a clinical diagnoses was not assessed. In a patient sample, risk estimations may indeed be more pronounced. Thus, further research needs to examine different risk perception components with a clinical sample. Furthermore, we did not control for other mental disorders or conditions that might have affected risk perception in our sample. Thus, future research should control for these differences in more standardized research designs. A further point refers to the fear domains we chose. Our selection was based on fear domains that are common in clinical samples (Hofmann et al., 2008) and that are comparable in terms of their evolutionary basis but often specifically feared in clinical samples. As a reference category, we chose generalized worries as they contain different daily fear triggers and could be contrasted against the animal fears (Meta-cognitions questionnaire; Cartwright-Hatton and Wells, 1997; Worry domains questionnaire: Tallis and de Silva, 1992). These fear domains did not build an exhaustive selection of fear areas and participants were not asked about personal fear-eliciting situations. Asking participants about their risk assessments about the personally fear-relevant conditions could result in further bias. Finally, we did not seek to examine implicit biases in information processing. Thus, memory or attentional biases might have caused the differences between high and low fearfuls. Future research designs should capture both, implicit and explicit information processing, thus allowing conclusions about the implicit processes in high fearful and low fearful individuals.

However, our results provide a first insight in distinguishable components of risk estimation with a large sample and a variety of different fears. Along with the good statistical power and the methodical as well as theoretical foundation for the measures of risk estimations.

Our findings are of special interest as cognitive interventions are often derived from cognitive models. These models also include elements of maladaptive risk evaluation. However, it has not been clear, what specific component of risk evaluation is actually biased in high fearful individuals. Importantly, the anticipation of negative outcomes has been found to be one of the main drivers of avoidance behavior in anxiety disorders (see Bublatzky et al., 2017; Pittig et al., 2018). Even in the absence of aversive experience, threat expectations may remain stable (Bublatzky et al., 2014) especially if they are not adequately addressed by therapeutic interventions. At the same time, perceptions of threat have been shown to be accessible to social instructions (Bublatzky et al., 2014). Therefore, cognitive models should include components of risk estimations in further detail and make predictions of their effect on avoidance behavior.

In addition, we have previously suggested that risk assessments should be more systematically targeted in the treatment of anxiety disorders (Alpers, 2010). The present findings provide further insight into the specific cognitions, which influence subsequent avoidance behavior; exaggerated assessments of aversive events may be a relevant target for therapeutic interventions. Of course, individual case analysis is needed to identify the biased component of risk estimations in each patient in order to adequately address the maladaptive mechanism.

## Data Availability

The datasets analyzed for this study can be found in the MADATA (University of Mannheim) Research Data Repository (doi: 10.7801/282).

## Ethics Statement

This study was carried out in accordance with the recommendations of the ethics committee of the University of Mannheim. Before providing any data, all participants were informed about the study purpose and the anonymous nature of the data collection. They were informed that the questions addressed their emotional experience with a variety of common situations but that these would not impose any substantial burden on them. They were then asked to explicitly indicate their consent by actively checking off on a box. People who did not consent at this time were not granted access to the questionnaire. All the participants who provided data in the online questionnaire remained completely anonymous, as the researchers did not save any personal data outside of the questionnaire. If the participants decided that they wanted to participate in a lottery after completion of the questionnaire, they were invited to send their email address to the researchers. This information was not linked to the data in any way.

## Author Contributions

KH and GA contributed to the following: design and implementation of the research, analysis of the results, and writing of the manuscript.

## Conflict of Interest Statement

The authors declare that the research was conducted in the absence of any commercial or financial relationships that could be construed as a potential conflict of interest.
