# Supplementary material for: What’s the Risk? Fearful Individuals Generally Overestimate Negative Outcomes and They Dread Outcomes of Specific Events
Source: Front Psychol. 2019 Jul 30;10:1676. doi: 10.3389/fpsyg.2019.01676 (PMC6682660; doi:10.3389/fpsyg.2019.01676)
Supplement: Supplementary file 1 [file Table_1.DOCX]

Supplementary Analyses

# Samples for the factor analyses

For the following factor analyses we split the dataset in two halves in a randomized manner with 315 participants being assigned to each. With one half (*M* = 30.21, *SD* = 10.28, Range = 16‒62, 76.5% females) we conducted an exploratory, with the other (*M* = 30.02, *SD* = 10.34, Range = 17‒66, 76.8% females) a confirmatory factor analysis to explore and confirm the data structure as well as to examine the construct validity. Questionnaire and demographic data are presented in Table S1.

Table S1. *Demographics and questionnaire data of the two samples.*

|  | Exploratory factor analysis | Confirmatory  analysis | *t/ χ^2^* | *p* |
| --- | --- | --- | --- | --- |
| n | 315 | 315 |  |  |
| Age | 30.21 *(10.28)* | 30.02 (*10.34*) | 0.32 ^a^ | .816 |
| Sex = Female | 241 *(76.5%)* | 242 *(76.8%)* | .01 ^b^ | .925 |
| Depression (BDI) | 30.79*(9.60)* | 31.44 *(9.88)* | -.84 ^a^ | .401 |
| State anxiety (STAI-S) | 39.12 *(11.60)* | 40.53 *(11.87)* | -1.50 ^a^ | .133 |
| Trait anxiety (STAI-T) | 40.83*(11.82)* | 41.86 *(12.61)* | -1.64^a^ | .245 |
| Optimism (LOT-R) | 22.71 *(4.55)* | 22.02 *(4.69)* | 1.90 ^a^ | .058 |
| Fear of spiders (FSQ) | 17.46 *(24.25)* | 18.27 *(24.23)* | -.42 ^a^ | .673 |
| Fear of snakes (SNAQ) | 7.23 *(6.41)* | 6.88 *(5.65)* | .73 ^a^ | .465 |
| Worry tendencies (PSWQ) | 45.23 *(12.86)* | 47.21 *(13.91)* | -1.87 ^a^ | .062 |

*Note.* Means (and standard deviations) separately for the exploratory and confirmatory factor analyses, for spider fearful and non-fearful, for snake fearful and non-fearful participants as well as for worriers and non-worriers. n = Number of participants; BDI = Beck Depression Inventory (Beck et al., 1996); STAI-S and -T = State and trait version of the State-Trait Anxiety Inventory (Laux et al., 1981; Spielberger et al., 1983) ; LOT-R = revision of the Life-Orientation-Test (Glaesmer, Hoyer, Klotsche & Herberg, 2008); FSQ = Fear of Spiders Questionnaire (Rinck et al., 2002; Szymanski & O’Donohue, 1995); SNAQ = Snake Questionnaire (Klorman, Weerts, Hastings, Melamed & Lang, 1974; Miltner, Gutberlet & Weiss, 2005); PSWQ = Penn State Worry Questionnaire (Meyer, Miller, Metzger & Borkovec, 1990) ; ^a^ *t* score for group comparison. ^b^ *χ^2^* score for gender ration comparison.

# Development of two risk questionnaires

To assess the risk estimations, we developed two questionnaires for measuring probability estimations of an encounter of specific events and for the negative estimated probability of negative consequences of such encounters, the *Risk of fear-relevant encounter* (REQ) and *Risk of negative outcome questionnaire* (RNOQ). Through an iterative process with professionals in the area of clinical and biological psychology and psychotherapy, we generated an item pool of 24 items for each questionnaire.

The resulting 48 items (24 for the REQ and 24 for the RNOQ) were rated on a 1 – 7 Likert-scale (‘very unlikely’ to ‘very likely’) of how likely the event and the consequence of such an event is, respectively. This scaling was used based on a questionnaire developed by Nesse and Klaas (1994) which assessed the risk perceptions of patients with anxiety disorders.

# Statistical analyses

Exploratory factor analyses as well as reliability analyses were mostly run by the statistical software package SPSS 24 (SPSS Inc., 2016). The open source software R 3.4.0 (2017) was used for analyses with supposed latent constructs as the confirmatory factor analysis (CFA).

First we conducted an exploratory factor analysis (EFA) to further examine the structure of the REQ and RNOQ in the questionnaire data with one half of the data set as described above. To check the number of factors to be extracted we used the scree-test according to the course of the Eigen values in the scree-plot. In the next step we fixated the number of factors which lied above the salient point of the Eigen value course. We used a principal axis analysis as the method of extraction because we were interested in the underlying constructs rather than the reduction of data. As we had the assumption that the factors should be related to each other we decided to apply a frequently used oblique rotation method, the promax rotation with Kaiser normalization to rotate the factors in a dependent matter. To have a balanced trade-off of between errors and biases we used the *καρρα* parameter on a value of 2 (Tataryn, Wood, & Gorsuch, 1999). According to recommendations for big sample sizes (Hair, Tatham, Anderson & Black, 1998), we set a cut-off of item loadings ≤ .3 to remove them. We also deleted items which loaded in the same range on two factors and items which did not match our content-related interpretation of the scale with their highest loading. After this procedure we ran a confirmatory factor analysis with the other half of the data set. For each questionnaire we planned to compare two competing models: One model with an assumed one factor structure and the other derived from the results of the prior conducted EFA. We used two types of absolute fit indices, the χ²-ratio test and the Root Mean Square Error of Approximation (RMSEA) to check the overall adequacy of the models and the convergence of the two indices. A value from .00‒2.00 indicates a good model fit and a value of 2.01‒3.00 of the χ²-ratio test an acceptable fit. For the RMSEA, values between .000‒.050 speak for a good model fit and values between .051‒.080 for an acceptable model fit (Hooper, Coughlan &Mullen, 2008; Moosbrugger & Kevala, 2012). To compare the two models we computed the Akaike information criterion (AIC) for unnested models. Face validity of the subscales were evaluated by the meaning of the scales. This assessed by the content of the items. We then undertook the two questionnaires and the resulting scales derived a reliability analysis from the factor analysis. This was to further assess the degree of accuracy oft he scales used which assessed the underlying construct.

# Exploration of the data structures

The scree-test for the REQ revealed a three-factor structure and a four-factor structure for the RNOQ. The courses of the Eigen values are shown in Figure 1.

**Figure 1.** Courses of the Eigen values of the items in the REQ (Risk of fear-relevant encounter questionnaire) and the RNOQ (Risk of negative encounter questionnaire). The black line marks the salient point above which the number of factors was chosen.

The three factors in the REQ explained 35.81% of the total variance in the data. The first factor can be named as ‘risk to encounter a spider’ because it only included items within the snake domain. The second factor could be interpreted in terms of the, ‘risk to encounter a snake’ as the factor loadings all laid in the negative range. The third factor could be named ‘risk to encounter everyday fear triggers’. This result confirmed our hypothesized data structure for this questionnaire and underlies the nature of domain-specifity for *Encounter* estimations. There was only one item (see Table S2) of the REQ (RE18) which had factor loadings ≤ .3 and it was removed of this factor.

The scree-test of the RNOQ revealed either a one- or four-factor solution according to the salient points. We chose the four-factor structure due to the larger fraction of explained variance (54.51 %) and due to content-related considerations as we assumed a minimum structure of three factors. According to their content these factors can be interpreted as ‘Danger-based fear (Spiders/Snakes)’, ‘Anxiety-based spider fear’, ‘Anxiety-based snake fear’ and ‘Catastrophizing’, respectively. Three items of the RNOQ (RNO06, RNO15, RNO23) loaded in the same range on two factors. There was one item (RNO10) which loaded most on a factor that did not match with our interpretation of it (‘Anxiety-based spider fear’). This was therefore removed, too.

Table S3 shows the pattern matrices of the RNOQ with the factor loadings of the single items on the respecting factors.

Table S2. *Factor loadings of the pattern matrix of the scales of the REQ.*

|  | Principal axis factor analysis  (*N* = 315) | | | |
| --- | --- | --- | --- | --- |
| Scale and assigned items | | 1 | 2 | 3 |
| Spider-encounter  You see a spider in an office building. (RE01) | | **.641** | -.034 | .036 |
| You see a spider in a city park. (RE05) | | **.741** | .055 | -.004 |
| You encounter a spider in a forest area. (RE07) | | **.778** | .012 | -.067 |
| You discover a spider in a restaurant. (RE09) | | **.553** | .184 | .162 |
| You see a spider in your home. (RE11) | | **.566** | -.089 | .129 |
| You encounter a spider in the attic. (RE14) | | **.633** | .009 | -.015 |
| You face a spider in the cellar. (RE22) | | **.620** | -.027 | .069 |
| Snake-encounter  You encounter a snake in the attic. (RE03) | | .030 | **.454** | .044 |
| You encounter a snake in fruits you bought in a supermarket. (RE06) | | -.071 | **.535** | .096 |
| You discover a snake in a restaurant. (RE12) | | -.032 | **.646** | .058 |
| You see a snake in your home. (RE15) | | -.002 | **.578** | .043 |
| You see a snake in a city park. (RE17) | | .159 | **.667** | -.053 |
| You see a snake in an office building. (RE20) | | -.035 | **.633** | .002 |
| You encounter a snake in a forest area.. (RE21) | | .249 | **.476** | -.041 |
| You face a snake in the cellar. (RE24) | | -.078 | **.755** | -.018 |
| Encounter everyday fear triggers  A family member has a traffic accident. (RE02) | | -.025 | .168 | **.401** |
| You have a personal argument with your friend. (RE04) | | .171 | -.076 | **.459** |
| You do accidentally not pay an invoice. (RE08) | | .054 | .034 | **.570** |
| You forget to turn off the stove after leaving your home. (RE10) | | .052 | .234 | **.460** |
| You are concerned a lot. (RE13) | | .083 | -.091 | **.354** |
| You forget an important professional appointment. (RE16) | | -.042 | .001 | **.634** |
| You accidentally overdraw your account. (RE19) | | .010 | .101 | **.469** |
| You encounter a discolored birthmark on your skin. (RE23) | | .126 | .107 | **.403** |
| Excluded item  You encounter a spider in fruits you bought in a supermarket. (RE18) | | .257 | .208 | .207 |

*Note.* Loadings in bold indicate the scale the items were assigned to.

Table S3*. Factor loadings of the pattern matrix of the scales of the RNOQ.*

|  | Principal axis factor analysis  (*N* = 315) | | | |
| --- | --- | --- | --- | --- |
| Item | 1 | 2 | 3 | 4 |
| Danger-based fear (spiders/snakes) |  |  |  |  |
| If you encounter a snake it will creep on your body. (RNO02) | **.535** | .099 | .278 | -.063 |
| If you see a spider it will kill you. (RNO08) | **.610** | .027 | .044 | -.062 |
| If you see a snake it will kill you. (RNO12) | **.714** | -.140 | .266 | .078 |
| If you see a snake it will follow you. (RNO13) | **.611** | -.023 | .384 | -.004 |
| If you encounter a spider it will bite you. (RNO17) | **.510** | .345 | -.074 | .159 |
| If you face a snake it will attack you. ( RNO18) | **.550** | -.023 | .393 | .110 |
| Anxiety-based spider fear  If you see a spider you will be frightened a lot. (RNO01) | -.111 | **.749** | .251 | .050 |
| If you discover a spider you will lose control over yourself. (RNO03) | -.016 | **.887** | .130 | .000 |
| If you discover a spider you will panic. (RNO22) | -.033 | **.853** | .195 | .009 |
| Anxiety-based snake fear  If you discover a snake you will panic. (RNO05) | .113 | .069 | **.864** | -.015 |
| If you discover a snake you will lose control over yourself. (RNO09) | .220 | .108 | **.757** | -.046 |
| If you see a snake you will be frightened a lot. (RNO20) | .056 | .052 | **.773** | .065 |
| General catastrophizing  If you encounter a discolored birthmark on your skin it will be skin cancer. (RNO04) | -.039 | .032 | .174 | **.456** |
| If a family member has a traffic accident he/she will lose his/her life. (RNO07) | .013 | -.046 | .202 | **.532** |
| If you have a personal argument with a friend he/she will denounce friendship.(RNO11) | -.001 | .060 | -.101 | **.512** |
| If you do accidentally not pay an invoice it will lead to a legal action. (RNO14) | .165 | .026 | -.043 | **.519** |
| If you forget to turn off the stove after leaving home it will cause a fire. (RNO16) | .016 | .026 | .087 | **.562** |
| If you are concerned a lot you will panic. (RNO19) | -.055 | .198 | .307 | **.447** |
| If you forget an important professional appointment you will be fired. (RNO21) | .165 | .036 | .009 | **.635** |
| If you accidentally overdraw your account you will be in need due to financial affairs. (RNO24) | -.011 | .093 | -.009 | **.696** |
| Excluded items |  |  |  |  |
| If you encounter a snake it will bite you. (RNO06) | .500 | .042 | .432 | .116 |
| If you encounter a spider it will scuttle on your body. (RNO10) | .281 | .438 | -.014 | .156 |
| If you see a spider it will follow you. (RNO15) | .476 | .545 | -.167 | .027 |
| If you face a spider it will attack you. (RNO23) | .561 | .552 | -.198 | .045 |

*Note.* Loadings in bold indicate the scale the items were assigned to.

# Confirmation of the data structures

To further confirm the data structure of the REQ we compared two competing models. Model 1 was a one-factor model with 23 items which were supposed to assess one latent structure. Model 2 was the prior assumed three-factor model with ‘risk to encounter a spider’, ‘risk to encounter a snake’ and the ‘risk to encounter everyday fear triggers’ as the three scales. Model 1 fitted the data poorly, *χ²*/df = 71.32, RMSEA = 0.117. However, the supposed Model 2 revealed an acceptable to good fit for the underlying data, χ²/df = 2.96, RMSEA = 0.079. Comparing the two competing untested models confirmed the assumption of the absolute fit indices: the three-factor model explained the data better, AIC = 770.304, than the one-factor model, AIC = 1316.676.

For the RNOQ we compared a one-factor model with all 20 items to assess one latent construct (Model 1) with the assumed four-factor model (Model 2) of the prior EFA. As for the REQ, Model 1 fitted the data only poorly, χ²/df = 9.95, RMSEA = 0.169, whereas Model 2 showed a poor to acceptable fit of the data, χ²/df = 3.14, RMSEA = 0.083, and even better, AIC = 607.215, than Model 1, AIC = 1771.330.

In sum, the three-factor model of the REQ with the scales ‘risk to encounter a spider’, ‘risk to encounter a snake’ and the ‘risk to encounter everyday fear triggers’, and the four-factor model of the RNOQ with the supposed scales ‘Danger-based anxiety (Spiders/Snakes)’, ‘Anxiety-based spider fear’, ‘Anxiety-based snake fear’ and ‘General catastrophizing’ revealed acceptable fits according to the data and were even better than the competing one-factor models. So they were used in further correlation analyses.

# Reliability analyses

In order to evaluate the accuracy of the items for their belonging scale, reliability analyses with the whole sample (*N* = 630) were conducted. For a measure of a personality facet rather than a measure of performance, the REQ and RNOQ revealed very good to excellent internal consistencies, REQ *α* = .83, and the RNOQ *α* = .88, respectively. Also for the scales of the REQ Cronbach‘s *α* showed good to very good values, ‘risk to encounter a spider’ *α* = .84, ‘risk to encounter a snake’ *α* = .78, and the ‘risk to encounter everyday fear triggers’ *α* = .72. This was similar for the scales of the RNOQ, ‘Danger-based anxiety (Spiders/Snakes)’ *α* = .82, ‘Anxiety-based spider fear’ *α* = .91, ‘Anxiety-based snake fear’ *α* = .89, and ‘General catastrophizing’ *α* = .79.

What is more, all items revealed sufficient discriminatory power for the REQ (Range = .29‒.61), and the RNOQ, (Range = .40‒.87), according to their related scales. Thus, the prior decided assignment of the items to the scales was further confirmed. For the complete reliability statistics and item characteristics see Table S4 to S6.

**7 Differentiation of risk estimations**

**7.1 Intra-correlations within risk estimations**

The correlation analyses within the *Encounter domains* revealed significant correlations between the *Encounter domain* ‘Spider’ and ‘Snake’, *r* = .22, *p* < .001, as well as between ‘Spider’ and ‘Everyday fear triggers’, *r* = .37, *p* < .001. There was also a significant relationship between the *Encounter domain* ‘Snake’ and ‘Everyday fear triggers’, *r* = .27, *p* < .001. Thus, risk estimations of encountering different fear-relevant stimuli were moderately correlated.

For the risk estimation of the different *Outcome domains*, we found significant moderate to high relationships between all *Outcome domains*, *r*s ≥ .32, all *p*s < .001.

Thus, risk estimations of encountering different fear-relevant stimuli were moderately correlated among each other, and risk estimations of negative outcomes of such encounters revealed moderately to high relationships.

**7.2 Inter-correlations between risk estimations**

The correlation analyses for the total scores between the two questionnaires first showed a significant relationship of a medium, *r* = .26, *p* < .001. This finding revealed that they assess both related, and two different latent dimensions.

Concerning the inter-correlations between the scales of the two questionnaires, the *Encounter domain* estimated ‘Snake’ was significantly correlated with the *Outcome domain* ‘Danger-based anxiety’ of fear-related animals, *r* = .27, *p* < .001, and with the scale ‘General catastrophizing’, *r* = .19, *p* < .001. The risk estimations of the Encounter domain ‘Everyday fear triggers’ were correlated with all negative outcome domains, all *r*s ≥ .14, all *p*s < .001. All other relationships did not turn out to be significant, all *r*s ≤ .08, all *p*s ≥ .055.

In sum, the inter-correlational pattern of the two questionnaires indicated that risk estimations of fear-relevant encounters and negative outcomes reflected are not the same; they measure different constructs (weak relationship between total scales and the non-significant to medium correlations between subscales).

Table S4. *Item characteristics of the final REQ questionnaire.*

|  | Mean (*SD*) | Corrected item scale correlations |
| --- | --- | --- |
|  | *N* = 630 | |
| Spider-encounter | 76.60 (*16.26*) |  |
| You see a spider in an office building. (RE01) | 10.79 (*3.34*) | .57 |
| You see a spider in a city park. (RE05) | 11.20 (*3.31*) | .62 |
| You encounter a spider in a forest area. (RE07) | 11.54 (*3.13*) | .64 |
| You discover a spider in a restaurant. (RE09) | 8.59 (*3.46*) | .55 |
| You see a spider in your home. (RE11) | 12.43 (*2.65*) | .60 |
| You encounter a spider in the attic. (RE14) | 10.18 (*3.97*) | .55 |
| You face a spider in the cellar. (RE22) | 11.87 (*2.90*) | .61 |
| Snake-encounter | 31.52 (*12.76*) |  |
| You encounter a snake in the attic. (RE03) | 3.88 (*2.90*) | .38 |
| You encounter a snake in fruits you bought in a supermarket. (RE06) | 3.30 (2.17) | .39 |
| You discover a snake in a restaurant. (RE12) | 3.46 (*2.29*) | .54 |
| You see a snake in your home. (RE15) | 3.30 (*2.53*) | .47 |
| You see a snake in a city park. (RE17) | 5.08 (*2.91*) | .60 |
| You see a snake in an office building. (RE20) | 2.99 (*2.20*) | .53 |
| You encounter a snake in a forest area.. (RE21) | 6.39 (*3.16*) | .46 |
| You face a snake in the cellar. (RE24) | 3.11 (*1.91*) | .61 |
| Encounter everyday fear triggers | 48.97 (*13.82*) |  |
| A family member has a traffic accident. (RE02) | 6.87 (*2.51*) | .37 |
| You have a personal argument with your friend. (RE04) | 9.79 (*3.10*) | .41 |
| You do accidentally not pay an invoice. (RE08) | 7.16 (*3.64*) | .53 |
| You forget to turn off the stove after leaving your home. (RE10) | 4.99 (*2.95*) | .43 |
| You are concerned a lot. (RE13) | 10.58 (3.38) | .29 |
| You forget an important professional appointment. (RE16) | 6.30 (*3.20*) | .52 |
| You accidentally overdraw your account. (RE19) | 6.33 (*3.94*) | .42 |
| You encounter a discolored birthmark on your skin. (RE23) | 7.53 (*3.01*) | .39 |

*Note.* Means (and standard deviations) and corrected item scale correlations of the merged items of the REQ. Each item was rated using a 7-point-likert scale. The ratings for the two time episodes were summed up so there was a range from 2– 14 for the means.

Table S5. *Item characteristics of the final RNOQ questionnaire.*

|  | Mean (*SD*) | Corrected item scale correlations | |
| --- | --- | --- | --- |
| Item | *N* = 630 | | |
| Danger-based fear (Spiders/Snakes) | 6.04 (*7.75*) | |  |
| If you encounter a snake it will creep on your body. (RNO02) | 1.58 *(1.07)* | | .53 |
| If you see a spider it will kill you. (RNO08) | 1.18 (*0.67*) | | .40 |
| If you see a snake it will kill you. (RNO12) | 1.42 (*0.89*) | | .69 |
| If you see a snake it will follow you. (RNO13) | 1.56 *(1.02)* | | .70 |
| If you encounter a spider it will bite you. (RNO17) | 1.40 (*0.89*) | | .54 |
| If you face a snake it will attack you. ( RNO18) | 1.84 *(1.19)* | | .67 |
| Panic of spiders | 7.94 (*5.08*) | |  |
| If you see a spider you will be frightened a lot. (RNO01) | 3.54 (*2.04*) | | .79 |
| If you discover a spider you will lose control over yourself. (RNO03) | 2.01 (*1.58*) | | .83 |
| If you discover a spider you will panic. (RNO22) | 2.39 (*1.88*) | | .87 |
| Anxiety-based snake fear | 8.01 (*4.89*) | |  |
| If you discover a snake you will panic. (RNO05) | 2.71 (*1.81*) | | .85 |
| If you discover a snake you will lose control over yourself. (RNO09) | 2.03 (*1.56*) | | .77 |
| If you see a snake you will be frightened a lot. (RNO20) | 3.28 (*2.00*) | | .75 |
| General catastrophizing | 21.36 (*7.29*) | |  |
| If you encounter a discolored birthmark on your skin it will be skin cancer. (RNO04) | 2.95 (*1.29*) | | .43 |
| If a family member has a traffic accident he/she will lose his/her life. (RNO07) | 2.86 (*1.26*) | | .52 |
| If you have a personal argument with a friend he/she will denounce friendship.(RNO11) | 2.40 (*1.25*) | | .41 |
| If you do accidentally not pay an invoice it will lead to a legal action. (RNO14) | 1.68 (*1.08*) | | .50 |
| If you forget to turn off the stove after leaving home it will cause a fire. (RNO16) | 3.15 (*1.57*) | | .51 |
| If you are concerned a lot you will panic. (RNO19) | 3.34 (*1.80*) | | .44 |
| If you forget an important professional appointment you will be fired. (RNO21) | 2.36 (*1.36*) | | .63 |
| If you accidentally overdraw your account you will be in need due to financial affairs. (RNO24) | 2.62 (*1.72*) | | .58 |

*Note.* Means (and standard deviations) and corrected item scale correlations of the items of the RNOQ. Each item was rated using a 7-point-likert scale.

Table S6. *Reliability coefficients of the different scales of the REQ and RNOQ*

| Scales | Cronbach´s α | Split-half |
| --- | --- | --- |
| REQ  (23 items) | .83 | .82 |
| Risk to encounter spiders  (7 items) | .84 | .78 |
| Risk to encounter snakes  (8 items) | .78 | .69 |
| Risk to encounter everyday fear triggers  (8 items) | .72 | .77 |
| RNOQ  (20 items) | .88 | .89 |
| Danger-based fear (Spiders/Snakes)  (6 items) | .82 | .82 |
| Anxiety-based spider fear  (3 items) | .91 | .94 |
| Anxiety-based snake fear  (3 items) | .89 | .89 |
| General Catastrophizing  (8 items) | .79 | .77 |

*Note.* Split-half coefficients represent the coefficient of the spearman-brown prophecy formula.
