# Supplementary material for: What’s the Risk? Fearful Individuals Generally Overestimate Negative Outcomes and They Dread Outcomes of Specific Events
Source: Front Psychol. 2019 Jul 30;10:1676. doi: 10.3389/fpsyg.2019.01676 (PMC6682660; doi:10.3389/fpsyg.2019.01676)
Supplement: Supplementary file 2 [file Table_2.DOCX]

Supplementary Tables and Figures

# Supplementary Table

Table S1. *Correlation analyses between the questionnaire data.*

|  |  | *N* | Mean (*SD*) | 1 | 2 | 3 | 4 |
| --- | --- | --- | --- | --- | --- | --- | --- |
| 1 | Depression  (BDI) | 630 | 31.12 (*9.71*) | 1 |  |  |  |
| 2 | State anxiety  (STAI-S) | 630 | 39.83 (*11.74*) | .70** | 1 |  |  |
| 3 | Trait anxiety  (STAI-T) | 630 | 41.39 (*12.22*) | .79** | .78** | 1 |  |
| 4 | Optimism  (LOT-R) | 630 | 22.37 (*4.63*) | -.61** | -.57** | -.73** | 1 |
| 5 | Fear of spiders  (FSQ) | 630 | 17.87 (*24.23*) | .21** | .18** | .23** | -.18** |
| 6 | Fear of snakes  (SNAQ) | 630 | 7.06 (*6.04*) | .11** | .14** | .13** | -.08* |
| 7 | Worry tendencies (PSWQ) | 630 | 46.22 (*13.42*) | .59** | .60** | .80** | -.64** |
| 8 | REQ  (total) | 630 | 167.67 (*32.11*) | .13** | .13** | .13** | -.11** |
| 9 | REQ  (spider encounter) | 630 | 76.60 (*16.26*) | -.03 | -.04 | -.05 | .05 |
| 10 | REQ  (snake encounter) | 630 | 31.52 (*12.76*) | .09* | .07 | .06 | -.09* |
| 11 | REQ (encounter of everday fear triggers  ) | 630 | 59.56 (*15.15*) | .23** | .26** | .29** | -.21** |
| 12 | RNOQ  (total) | 630 | 46.29 (*15.91*) | .35** | .31** | .41** | -.38** |
| 13 | RNOQ (bodily threat by spiders/snakes) | 630 | 8.98 (*4.22*) | .15** | .13** | .17** | -.19** |
| 14 | RNOQ  (panic of spiders) | 630 | 7.94 (*5.08*) | .23** | .21** | .27** | -.22** |
| 15 | RNOQ  (panic of snakes) | 630 | 8.01 (*4.89*) | .11** | .11** | .14** | -.10*- |
| 16 | RCQ (generalized catastrophizing) | 630 | 21.36 (*7.29*) | .45** | .39** | .50** | -.49** |

*Note.* * correlation is significant at the 0.05 level (1-tailed) and ** correlation is significant at the 0.01 level (2-tailed). N = Number of participants; BDI = Beck Depression Inventory (Hautzinger et al., 2006; STAI-S and -T = State and trait version of the State-Trait Anxiety Inventory (Laux et al., 1981) ; LOT-R = revision of the Life-Orientation-Test (Glaesmer et al., 2008); FSQ = Fear of Spiders Questionnaire (Rinck et al., 2002); SNAQ = Snake Questionnaire (Klorman et al., 1974); PSWQ = Penn State Worry Questionnaire (Meyer et al., 1990).

# Supplementary Figures

**Figure S1.** Relationships between the FQS and the scales of the RNOQ. The lines mark the linear trend of the relationships.

**Figure S2.** Relationships between the SNAQ and the scales of the RNOQ. The lines mark the linear trend of the relationships.
